# Supplementary material for: Rethinking Suicide in Rural Australia: A study Protocol for Examining and Applying Knowledge of the Social Determinants to Improve Prevention in Non-Indigenous Populations
Source: Int J Environ Res Public Health. 2019 Aug 16;16(16):2944. doi: 10.3390/ijerph16162944 (PMC6719075; doi:10.3390/ijerph16162944)
Supplement: Supplementary file 1 [file ijerph-16-02944-s001.pdf]

Supplementary Table S1: Codebook for National Coronial Information System data

**SOCIODEMOGRAPHIC**				**CODE**
**Sex**	Male			1
	Female			2
	Unknown			99
**Marital Status**	Married (incl. de facto)			1
	Never married			2
	Separated			3
	Divorced			4
	Widowed			5
	Unknown			99
**Employment Status**	Employed			1
	Unemployed			2
	Student			3
	Retired/ [Disability] Pensioner			4
	Home duties			5
	Prisoner			6
	Other			98
	Unknown			99
**Indigenous Status**	Neither Aboriginal nor Torres Strait Islander			0
	Aboriginal and/or Torres Strait Islander			1
	Unknown			99
**CIRCUMSTANCES OF DEATH**				**CODE**
**Incident Location**	Home	Unspecified Type of Home		1
		Detached house		2
		Flat, Apartment, Terrace House		3
		Farmhouse		4
		Residential Caravan, Mobile Houseboat		5
		Granny flat, Bungalow		6
		Boarding house, Hostel		7
		Hut, Shanty, Humpy		8
	Industrial or Construction Area	Other Specified Commercial Area (Non-Recreational)		10
		Grain silo, Storage		11
		Construction Site		12
		Factory, Plant		13
		Shearing Shed		14
		Mine and Quarry		15
	Commercial Area	Unspecified		20

		Motel, Hotel		21
		Shop, Store		22
		Storage Facility		23
		Café, Pub		24
		Commercial Garage		25
		Office Building		26
	Recreational Area/Cultural Area or Public Building	Other Specified Recreational, Cultural Area, or Public Building		30
		Holiday Park, Campground		31
		Public Park		32
		Cemetery, Crematorium		33
		Public Religious Place		34
		Holiday Resort		35
		Off Road Park		36
	School/Educational Area	Unspecified		40
		School, University		41
		Student Accommodation		42
	Residential Institution Area	Unspecified		50
		Home for the Elderly, Retirement Village		51
		Police Station		52
		Prison, Youth Training or Detention Centre		53
		Military Institution		54
		Residential Care Facility		55
		Crisis Accommodation		56
	Medical Service Area	Unspecified		60
		Hospital		61
		Nursing Home		62
	Countryside	Unspecified Countryside		70
		Forest, Bushland		71
		Large Area of Water		72
		Area of Still Water		73
		Stream of Water		74
		Beach, Shore, Bank of a Body of Water		75
		Base of Precipice, Cliff		76
		Lookout, Viewpoint		77
		Remote or Undeveloped Place		78
	Farm or Other Place of Primary Production	Unspecified Farm or Other Place of Primary Production		80
		Paddock, Field		81

		Area for Raising or Care of Animals		82
		Area for Growing of Crops Combined with Raising/Care of Animals (Mixed Farming)		83
		Hobby Farm		84
		Timber Plantation		85
		Area for Growing of Crops, Market Gardening, Horticulture		86
	Transport Area/Public Highway/Freeway	Unspecified Public Highway, Road		90
		Roadway (Plus) Highway, Freeway		91
		Bush Track, Dirt Road		92
		Footpath		93
		Railway (Other Than Station)		94
		Public Transport Station/Facilities		95
		Rest Area, Parking Bay (PLUS) Parking Area		96
	Sports & Athletic Area	Other Specified Sports and Athletics Area		100
		Sporting Grounds (Outdoor)		101
		Other Specified Facilities		102
	Other Places of Occurrence	Unspecified		110
		Bridge		111
		Land Vehicle		112
		Wharf, Pier, Jetty		113
		Indigenous Community		114
	Other			98
	Unknown			99
**Death Location**	Incident Location			1
	Medical Facility			2
	Other Non-Incident Location			98
	Unknown			99
**Method**	Contact with Static Object (Jumping/Falling)			1
	Contact with Fire or Flame			2
	Drowning			3

	Exposure to Electric Current			4
	Hanging, Strangling			5
	Inhalation of Smoke			6
	Obstruction of Airway by Object Covering Mouth			7
	Puncture, Stabbing, Cutting, Severing			8
	Self-Immolation			9
	Poisoning by Multiple Substances	Alcohol and Antidepressants		10
		Alcohol and Amphetamines		11
		Alcohol and Analgesics		12
		Alcohol and Benzodiazepines		13
		Alcohol and Cannabis		14
		Alcohol and Opiates		15
		Amphetamines and Antidepressants		16
		Amphetamines and Opiates		17
		Analgesics		18
		Antidepressants		19
		Antidepressants and Analgesics		20
		Antidepressants and Benzodiazepines		21
		Antidepressants and Cannabis		22
		Antidepressants and Opiates		23
		Benzodiazepines and Analgesics		24
		Benzodiazepines and Opiates		25
		Benzodiazepines		26
		Other Specified or Unspecified Multiple Substances		27
	Poisoning by Solid, Gaseous and Liquid Substance - *pharmaceutical*	Analgesic, Antipyretic, Antirheumatic		30
		Amphetamine		31
		Anticonvulsant		32
		Antidepressants		33

		Benzodiazepine		34
		Cardiovascular drug		35
		Insulin		36
		Sedative, Hypnotic, Antipsychotic		37
		Other Specified or Unspecified Pharmaceutical Substance		38
	Poisoning by Solid, Gaseous and Liquid Substance – *non-pharmaceutical*	Cleaning Agent		40
		Helium		41
		LPG Gas, Natural Gas, Methane Gas, Propane Gas, Butane Gas		42
		Motor Vehicle Exhaust		43
		Nitrogen		44
		Other Sources of Carbon Monoxide		45
		Plant Food, Veterinary Product, Pesticide or Fertiliser		46
		Strychnine		47
		Alcohol		48
		Other Specified or Unspecified Non-pharmaceutical Substance		49
	Shot by firearm	Handgun		50
		Shotgun		51
		Rifle		52
		Other Unspecified Firearm		53
	Transport Injury - Pedestrian<br>Transport Injury – Vehicle Occupant	Heavy Transport Vehicle		60
		Light Transport Vehicle		61
		Train		62
		Other Specified or Unspecified Vehicle		63
		Heavy Transport Vehicle		70
		Light Transport Vehicle		71
		Other Specified or Unspecified Vehicle		72
	Other			98
	Unknown			99
**Presence of Alcohol and/or Other drugs at Time of Incident**		No		0
		Alcohol		1
		Alcohol and Antidepressants		2

		Alcohol and Amphetamines		3
		Alcohol and Analgesics		4
		Alcohol and Benzodiazepines		5
		Alcohol and Cannabis		6
		Alcohol and Opiates		7
		Amphetamines		8
		Amphetamines and Antidepressants		9
		Amphetamine and Cannabis		10
		Analgesic, Antipyretic, Antirheumatic		11
		Anticonvulsant		12
		Antidepressants		13
		Antidepressants and Analgesics		14
		Antidepressants and benzodiazepines		15
		Antidepressants and Cannabis		16
		Antidepressants and Opiates		17
		Benzodiazepines		18
		Benzodiazepines and Opiates		19
		Benzodiazepines and Cannabis		20
		Cannabis		21
		Cannabis and Ecstasy		22
		Sedative, Hypnotic, Antipsychotic		23
		Other Specified or Unspecified Substance(s)		24
		Other		98
		Unknown		99
**HEALTH STATUS**				**CODE**
**Diagnosed Mental Illness**	No			0
	Yes	Mood Disorder	Depression	1
			Bipolar	2
		Anxiety Disorder		3
		Psychotic Disorder		4
		Eating Disorder		5
		Substance Abuse Disorder		6
		Personality Disorder		7

		Trauma-related disorder		8
		Psychiatric Comorbidity		9
		Suspected Mental Health Problem		10
		Other		98
		Unknown		99
**Treatment**	No			0
	Yes	Medication		1
		Psychotherapy/Counselling		2
		Medication and Counselling/Therapy		3
		Other		98
		Unknown		99
**Reported Physical Illness**	Injury/Disability	No		0
		Yes		1
		Unknown		99
	Cardiovascular Disease	No		0
		Yes		1
		Unknown		99
	Chronic Kidney Disease	No		0
		Yes		1
		Unknown		99
	Respiratory Disease	No		0
		Yes		1
		Unknown		99
	Musculoskeletal Conditions	No		0
		Yes		1
		Unknown		99
	Cancer	No		0
		Yes		1
		Unknown		99
	Dementia	No		0
		Yes		1
		Unknown		99
	Other	No		0
		Yes		1
		Unknown		99
**Contact with Health Services**	Community Treatment Order/Continuing care Order	No		0
		Yes		1
		Unknown		99
	Inpatient Mental Health	No		0
		Yes		1
		Unknown		99
	Proximity of Care Episode	None		0
		< 1 Week		1
		1-6 Weeks		2

		6 Weeks to 6 Months		3
		6 Months to 1 Year		4
		>1 Year		5
		Unknown		99
	Emergency Department	No		0
		Yes		1
		Unknown		99
	Proximity of Care Episode	None		0
		< 1 Week		1
		1-6 Weeks		2
		6 Weeks to 6 Months		3
		6 Months to 1 Year		4
		>1 Year		5
		Unknown		99
	Psychiatrist/Psychologist	No		0
		Yes		1
		Unknown		99
	Proximity of Care Episode	None		0
		< 1 Week		1
		1-6 Weeks		2
		6 Weeks to 6 Months		3
		6 Months to 1 Year		4
		>1 Year		5
		Unknown		99
	Community Mental Health	No		0
		Yes		1
		Unknown		99
	Proximity of Care Episode	None		0
		< 1 Week		1
		1-6 Weeks		2
		6 Weeks to 6 Months		3
		6 Months to 1 Year		4
		>1 Year		5
		Unknown		99
	Drug and Alcohol Services	No		0
		Yes		1
		Unknown		99
	Proximity of Care Episode	None		0
		< 1 Week		1
		1-6 Weeks		2
		6 Weeks to 6 Months		3
		6 Months to 1 Year		4
		>1 Year		5
		Unknown		99
	Primary Care	No		0
		Yes		1

		Unknown		99
	Proximity of Care Episode	None		0
		< 1 Week		1
		1-6 Weeks		2
		6 Weeks to 6 Months		3
		6 Months to 1 Year		4
		>1 Year		5
		Unknown		99
	Allied Health	No		0
		Yes		1
		Unknown		99
	Proximity of Care Episode	None		0
		< 1 Week		1
		1-6 Weeks		2
		6 Weeks to 6 Months		3
		6 Months to 1 Year		4
		>1 Year		5
		Unknown		99
	Tertiary Care (Physical health)	No		0
		Yes		1
		Unknown		99
	Proximity of Care episode	None		0
		< 1 Week		1
		1-6 Weeks		2
		6 Weeks to 6 Months		3
		6 Months to 1 Year		4
		>1 Year		5
		Unknown		99
	Palliative Care	No		0
		Yes		1
		Unknown		99
	Other	No		0
		Yes		1
		Unknown		99
**Contact with Social Services**	Family and Community Services	No		0
		Yes		1
		Unknown		99
	Centrelink [Social Security]	No		0
		Yes		1
		Unknown		99
	Dept. of Veterans Affairs	No		0
		Yes		1
		Unknown		99
	Criminal Justice	No		0
		Yes		1
		Unknown		99

	Housing	No		0
		Yes		1
		Unknown		99
	Crisis Line	No		0
		Yes		1
		Unknown		99
	Other	No		0
		Yes		1
		Unknown		99
**SOCIOECONOMIC AND PSYCHOSOCIAL CIRCUMSTANCES**				**CODE**
**Problems Related to Employment and Unemploy-ment**		No		0
		Unemployment		1
		Change of Job		2
		Threat of Job Loss		3
		Stressful Work Conditions		4
		Discord with Boss or Colleagues		5
		Sexual or Verbal Harassment/Bullying		6
		Injury/Workers Compensation		7
		Other/unspecified		98
		Unknown		99
**Problems Related to Housing and Economic Circumstances**		No		0
		Low Income		1
		Extreme Poverty		2
		Insufficient Welfare Support		3
		Debt		4
		Gambling		5
		Inadequate Housing		6
		Homelessness		7
		Housing Insecurity		8
		Discord with Neighbours, Lodgers, Landlord		9
		Eviction		10
		Other/unspecified		98
		Unknown		99
**Problems Related to Social Environment**		No		0
		Problems of Adjustment to Life-cycle Transitions		1
		Problems Related to Living Alone		2

		Acculturation Difficulty		3
		Social Exclusion and Rejection		4
		Target of Perceived Adverse Discrimination and Persecution		5
		Problems Related to Sexuality/Gender Identity		6
		Problems Related to Family Violence		7
		Other/unspecified		98
		Unknown		99
**Problems Related to Upbringing**		No		0
		Upbringing Away from Parents		1
		Problems Related to Alleged Sexual Abuse		2
		Problems Related to Alleged Physical Abuse		3
		Parent-Child Conflict		4
		Parent-Child Estrangement		5
		Problems Related to Child Custody		6
		Other/unspecified		98
		Unknown		99
**Other Problems Related to Primary Support Group incl. Family Circumstances**		No		0
		Problems in Relationship with Spouse or Partner		1
		Disruption of Family by Separation or Divorce		2
		Problems in Relationship with Children		3
		Problems in Relationship with In-Laws		4
		Death of Family Member or Friend	Non-suicide related death	5
			Suicide related death	6
		Dependent Relative or Friend Needing Care at Home		7

		Alcoholism and Drug Use in the Family		8
		Other/unspecified		98
		Unknown		99
**Problems Related to Education and Literacy**		No		0
		Educational Maladjustment and/or Discord with Teachers and Classmates		1
		Failed School Examinations		2
		Underachievement		3
		Other/unspecified		98
		Unknown		99
**Problems Related to Certain Psychosocial Circumstances**		No		0
		Problems Related to Pregnancy		1
		Problems Related to Multiparity		2
		Discord with Counsellors		3
		Conviction in Civil or Criminal Proceedings		4
		Imprisonment and incarceration		5
		Problems Related to Release from Prison		6
		Problems Related to Other Legal Circumstances		7
		Victim of Crime		8
		Problems Related to Addiction		9
		Exposure to Disaster, War & Other Hostilities		10
		Other/unspecified		98
		Unknown		99
